# Results From the Practice Accreditation Resident Reviewer Program (PARRP) Pilot: Educating Radiation Oncology Residents About Practice Accreditation

**DOI:** 10.7759/cureus.106072

**Published:** 2026-03-29

**Authors:** Niema B Razavian, Alexis N Schutz, Leslie Chang, Valerie Guth, Srinath Sundararaman, Rojymon Jacob, Kathryn Huber, Steven J Feigenberg, Jaroslaw T Hepel

**Affiliations:** 1 Radiation Oncology, Moffitt Cancer Center, Tampa, USA; 2 Radiation Oncology, Mercy Hospital, Oklahoma City, USA; 3 Radiation Oncology, University of Minnesota, Minneapolis, USA; 4 Accreditation, American College of Radiation Oncology, Jacksonville, USA; 5 Radiation Oncology, University of North Carolina (UNC) Health - Pardee Cancer Center, Hendersonville, USA; 6 Radiation Oncology, University of Mississippi Medical Center, Jackson, USA; 7 Radiation Oncology, Beth Israel Deaconess Medical Center, Harvard Medical School, Boston, USA; 8 Radiation Oncology, University of Pennsylvania, Philadelphia, USA; 9 Radiation Oncology, Brown University, Providence, USA

**Keywords:** medical education, medical resident education, pilot project, quality improvement, radiation oncology, radiation oncology education

## Abstract

Introduction: While instruction in patient safety and quality improvement is a core requirement for radiation oncology graduate medical education, many residents feel their training in this domain is inadequate. To address this discrepancy, we developed a mentorship program that allows residents to serve as junior reviewers within the American College of Radiation Oncology (ACRO) Practice Accreditation Program. Herein, we present the results of the pilot experience.

Methods: Participating residents served as junior reviewers with the ACRO accreditation program and completed four cycles of chart review assessing the quality of treatment documentation. During each cycle, residents were paired 1:1 with an experienced reviewer within the accreditation program. Both resident and attending independently reviewed the same set of charts and scored documentation quality using the same rubric. After the chart evaluation, the pairs met to discuss scoring and provide feedback. Residents and attendings were surveyed at the beginning and end of each cycle. Responses were scored using a modified Likert scale ranging from 1 (not at all confident/satisfied) to 7 (very confident/satisfied).

Results: A total of three residents and four attending mentors participated in this pilot. Participating residents were PGY-4s at study initiation and PGY-5s at completion. Attendings were disease-site experts with >2 years of practice-accreditation review experience. Across the four cycles of chart review, residents reviewed a total of 15-16 charts (3-4 charts per cycle). The median length of resident-mentor feedback meetings ranged from 31 to 60 min. Residents’ confidence in their chart-review abilities increased with each cycle of chart review as follows: pre-cycle confidence increased from a mean of 3 (range: 2-4) in cycle one to a mean of 5.3 (range: 5-7) in cycle four; while post-cycle confidence increased from a mean of 4 (range: 4-5) in cycle one to a score of 6 for all participants in cycle four. Residents’ perceived need for additional cycles of chart review decreased over time - from 100% in cycles one and two, to 0% in cycle four. Resident confidence in independent documentation review increased with completion of the program as follows: from pre-pilot mean of 1.7 (range: 1-2) to post-pilot mean of 6.7 (range: 6-7).

Conclusions: In this pilot study, we demonstrated that mentored chart review can improve resident confidence in practice accreditation. Resident confidence appeared to peak after four cycles of chart review. Structured mentorship programs such as the Practice Accreditation Resident Reviewer Program (PARRP) may help supplement radiation oncology education in patient safety and quality improvement.

## Introduction

Patient safety and quality improvement (PSQI) are an integral part of residency training in radiation oncology (RO). According to the Accreditation Council for Graduate Medical Education (ACGME), residency programs must ensure residents are educated on PSQI and assess their knowledge of this topic throughout training [1,2]. While programs have used multiple methods to teach PSQI, many residents feel their instruction in PSQI is inadequate [3,4].

Practice accreditation is a potential avenue for educating residents on PSQI. Accreditation is a voluntary process in which a RO facility’s treatment records are audited to ensure that safety and quality standards are followed. While the specifics of the audit vary by accreditation program, the process typically involves review of a facility’s medical and physics records [5-7]. Medical records reviews, for example, assess multiple aspects of RO practice, such as the history and physical, simulation, treatment planning, and treatment summary. After reviews are completed, facilities are provided with feedback on how documentation and practice standards can be improved.

To give RO residents hands-on experience in PSQI, we developed the Practice Accreditation Resident Reviewer Program (PARRP). In this program, RO residents serve as junior reviewers within the American College of Radiation Oncology (ACRO) Practice Accreditation Program and are mentored by attending ROs. Herein, we describe the results of the initial pilot PARRP, including both RO resident and attending perspectives on the experience.

## Materials and methods

**Figure 1:**
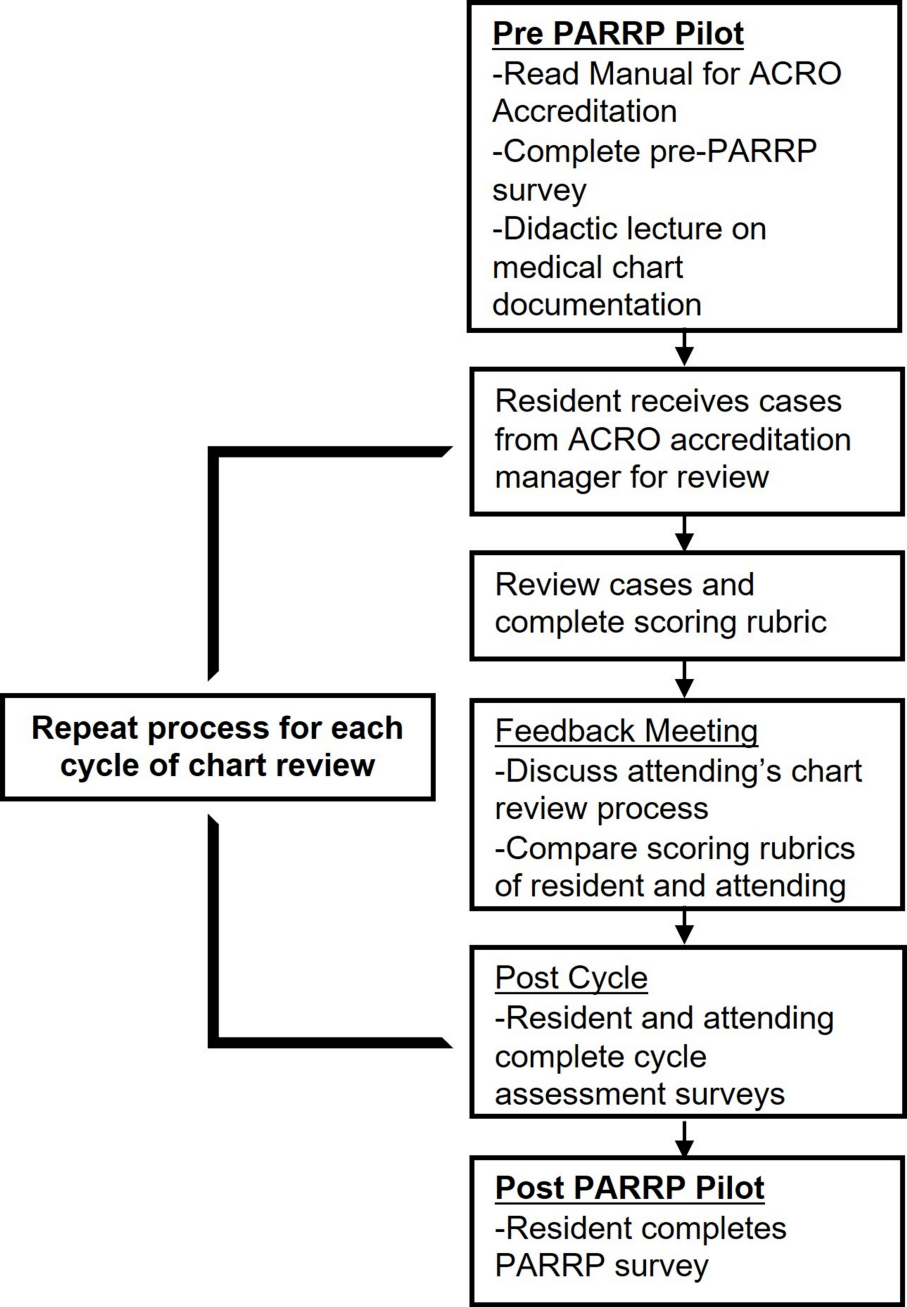
Overview of the PARRP pilot. PARRP: Practice Accreditation Resident Reviewer Program; ACRO: The American College of Radiation Oncology

An overview of the PARRP pilot is shown in Figure 1. Prior to starting the program, resident participants received instructional materials to gain familiarity with the accreditation process and scoring system. They were also given a didactic lecture regarding the importance of medical chart documentation and the various elements of documentation throughout the process of care specific to radiation therapy. At the beginning of each chart review cycle, residents and attending participants were paired and assigned the same cases based on the attendings’ disease site expertise to review. Both groups scored cases using the same ACRO accreditation program rubric, which was specific to each disease site. After scoring was completed, a feedback meeting was arranged between resident and attending pairs. For each feedback meeting, pairs were instructed to discuss approaches to case review and to compare completed scoring forms. The PARRP pilot was planned for a total of four chart review cycles. For each chart review cycle, each resident rotated with a different attending physician and scored charts from a different disease site.

Surveys were administered throughout the PARRP pilot (Figure 1). They were designed by the lead author and accreditation program director with input from attending physicians within the accreditation program. After all review cycles were completed, residents were given a post-pilot survey to assess their confidence across different domains of the accreditation process (tables in appendix). Questions about confidence and satisfaction were rated on a modified Likert scale ranging from 1 (not at all confident/satisfied) to 7 (very confident/satisfied). Surveys were administered by the accreditation manager using an online survey system. Data were locked for analysis until project completion. Given the limited size of the pilot project, survey data were summarized using descriptive statistics with mean (and range) values presented.

## Results

Participant baseline characteristics

A total of three residents and four attending ROs participated in the pilot PARRP (Table 1). In terms of training level, all residents were PGY-4s at the start of the pilot, and PGY-5s by the time of completion. Two of the resident participants were women. All residents had no prior experience with documentation review, and two had no formal training in patient safety and/or quality improvement. The mean (range) Likert score for perceived benefit of chart review mentorship prior to study initiation was 6 (5-7), indicating residents felt they would benefit from mentorship.

**Table 1:** Characteristics of PARRP pilot participants. PGY: post-graduate year; PSQI: patient safety and quality improvement; RO: radiation oncology; ACRO: American College of Radiation Oncology; PARRP: Practice Accreditation Resident Reviewer Program

Resident participants	Total (n=3)
PGY year	
PGY-4	3 (100%)
Gender	
Male	1 (33%)
Female	2 (66%)
Formal training in PSQI	
No	2 (66%)
Yes	1 (33%)
Attending participants	Total (n=4)
Time as an RO attending	
11-20 years	1 (25%)
20+ years	3 (75%)
Time as an ACRO accreditation faculty	
2-5 years	1 (25%)
6-10 years	2 (50%)
11+ years	1 (25%)
Frequency of resident interaction	
Never-rare	1 (25%)
Frequently	3 (75%)

Among attending participants, the majority (75%) had 20+ years of experience as an attending RO (Table 1). Most had six or more years of experience as an accreditation reviewer for their respective disease sites. Disease sites covered included thoracic, genitourinary, breast, and gastrointestinal malignancies. The majority (75%) of attending reviewers worked at academic institutions and were involved in resident education.

Timing of case reviews and feedback meetings

All residents completed the specified four cycles of chart review. With the exception of one resident in cycle 1, residents reviewed four cases per cycle (Table 2). Completion of post-cycle surveys was 92% for residents (11/12 surveys completed) and 75% for attendings (9/12 surveys completed). All residents completed the pre- and post-pilot surveys.

**Table 2:** Completion of chart reviews and surveys by residents and attending reviewers.

Chart review cycle	Number of charts reviewed per cycle (% residents)	Number of residents completing surveys per cycle (% residents)	Number of attending reviewers completing surveys per cycle (% attendings)
Cycle 1	3 (33%), 4 (66%)	3 (100%)	2 (66%)
Cycle 2	4 (100%)	2 (66%)	2 (66%)
Cycle 3	4 (100%)	3 (100%)	3 (100%)
Cycle 4	4 (100%)	3 (100%)	2 (66%)

**Figure 2:**
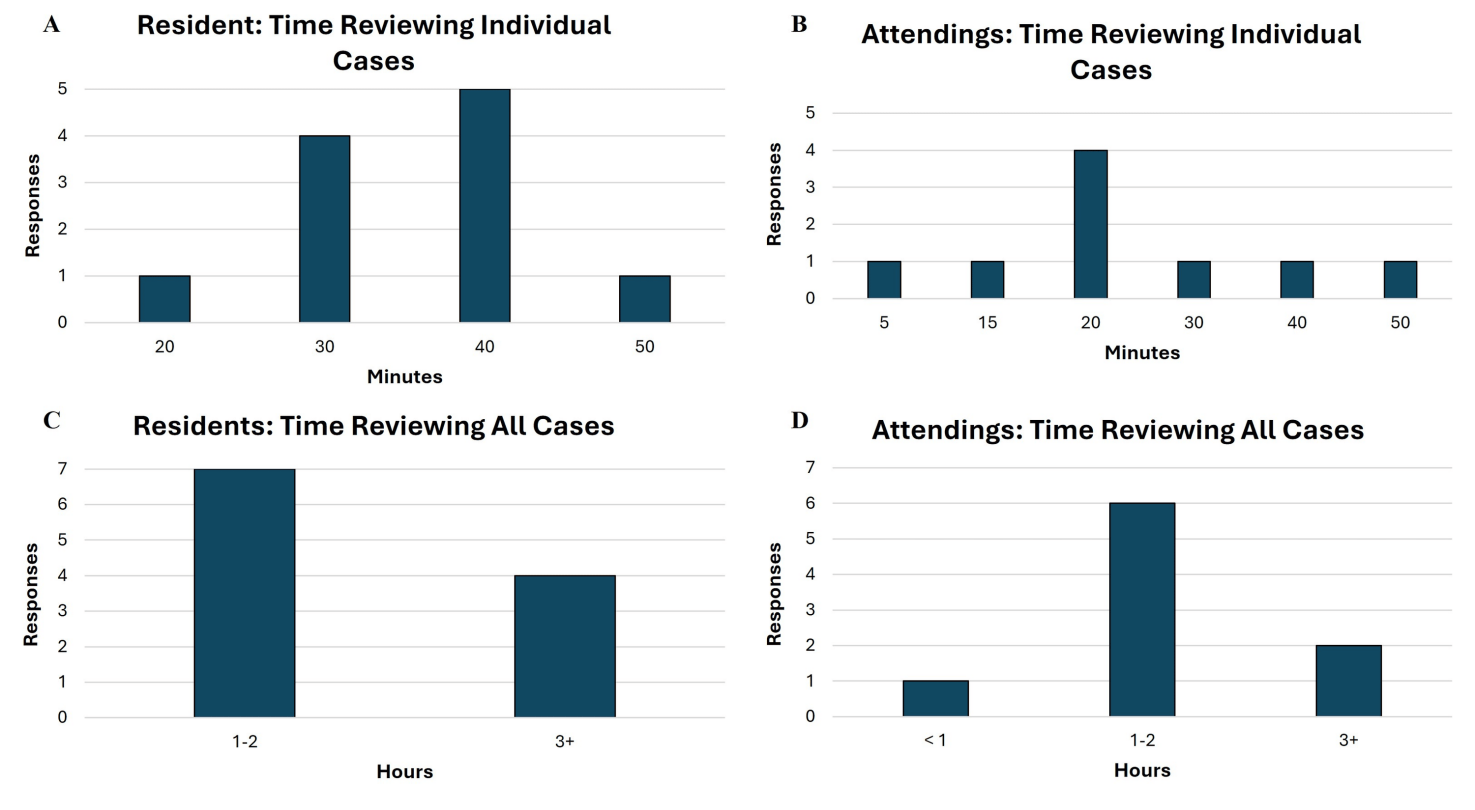
Time spent reviewing cases per cycle. Time spent reviewing by residents and attendings reviewing individual cases per cycle (A, B) and all cases per cycle (C, D).

Time spent reviewing cases by residents and attendings is displayed in Figures 2A, 2B. For both groups, review time for individual cases ranged from 5 to 50 minutes (Figure 2A, 2B). Compared with experienced reviewers, residents reported spending more time reviewing individual cases: in post-cycle surveys, 91% of cases reviewed by residents took 30 minutes or more, compared with 33% of cases reviewed by attendings. Among residents and attendings, the most frequently reported time to review all cases per cycle was 1-2 h (Figures 2C, 2D). A greater proportion of residents (36%) than attendings (22%) reported taking 3 or more hours to review all cases per cycle.

**Figure 3:**
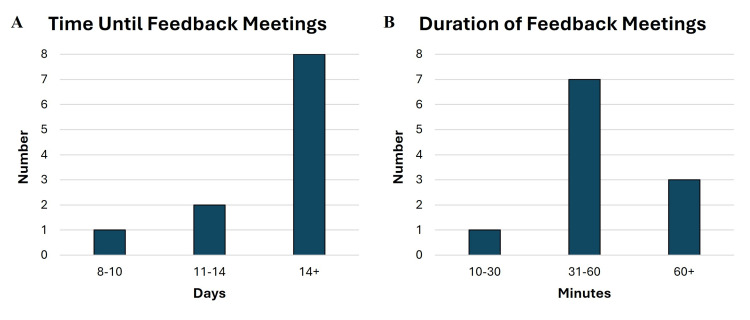
Timing and duration of feedback meetings. Time from completion of case review to feedback meetings (A) and duration of feedback meetings (B).

Feedback meetings between residents and attendings were completed during each cycle of chart review. Of the 12 feedback meetings, 11 were conducted virtually and one was conducted in person. Most meetings (73%) took place 14 or more days after residents completed case review (Figure 3A). The majority of meetings (64%) lasted between 31 and 60 minutes, with only one meeting lasting between 10 and 30 minutes (Figure 3B). 

Resident and attending perceptions after each cycle of chart review

**Figure 4:**
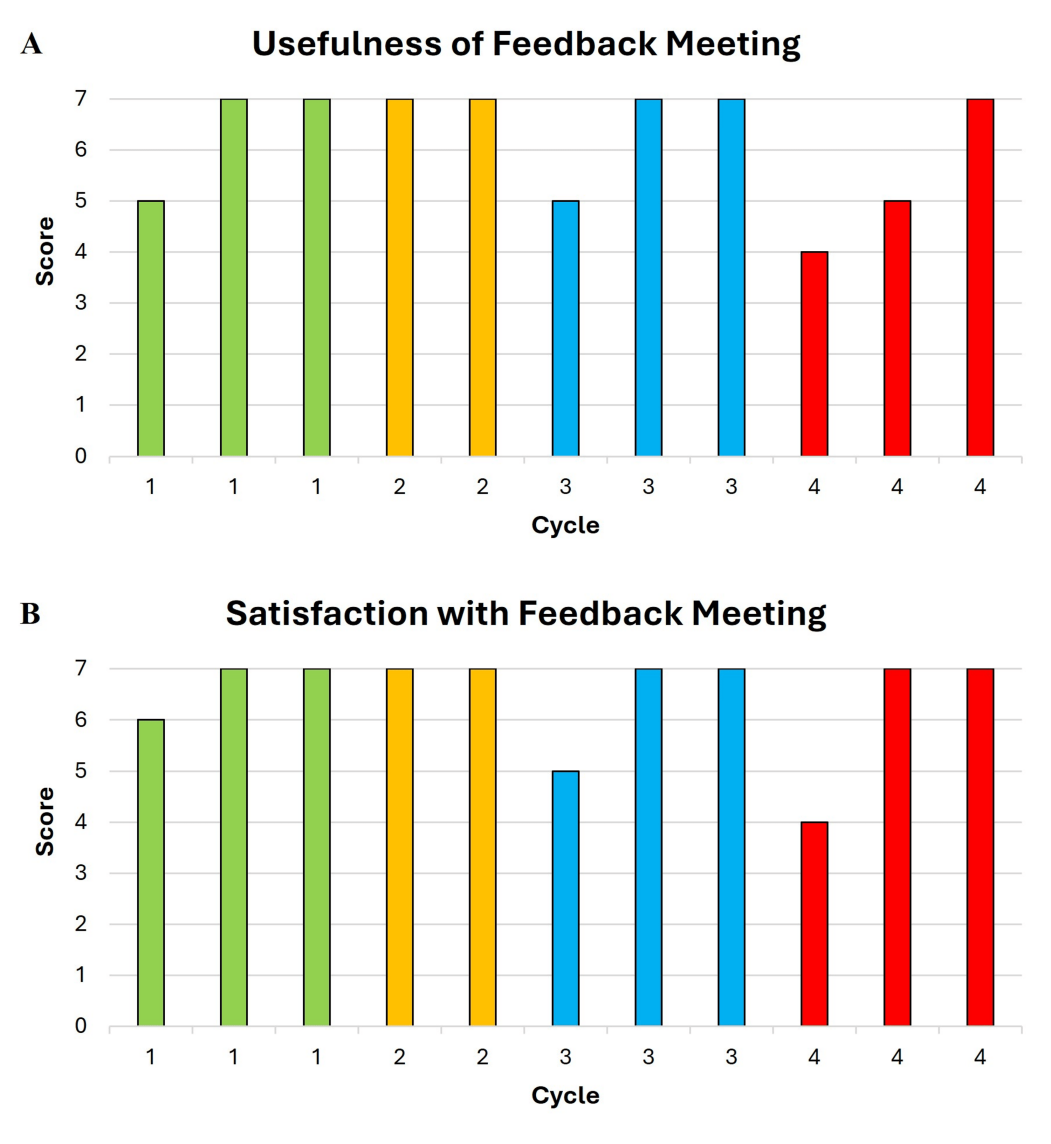
Resident perceptions on feedback meetings. Residents rated the usefulness and satisfaction of each feedback meeting (from 1 to 7). Each column represents one resident response and is arranged from lowest to highest score.

Resident perceptions of feedback meetings are displayed in Figures 4A, 4B. The mean score for the usefulness of feedback meetings was 6.3 (5-7), 6 (6), 6.3 (5-7), and 5.3 (4-7) in cycles 1-4, respectively (Figure 4A). Similarly, resident satisfaction with feedback meetings was rated 6.7 (6-7), 7 (7), 6.3 (5-7), and 6 (4-7) in cycles 1-4, respectively (Figure 4B).

**Figure 5:**
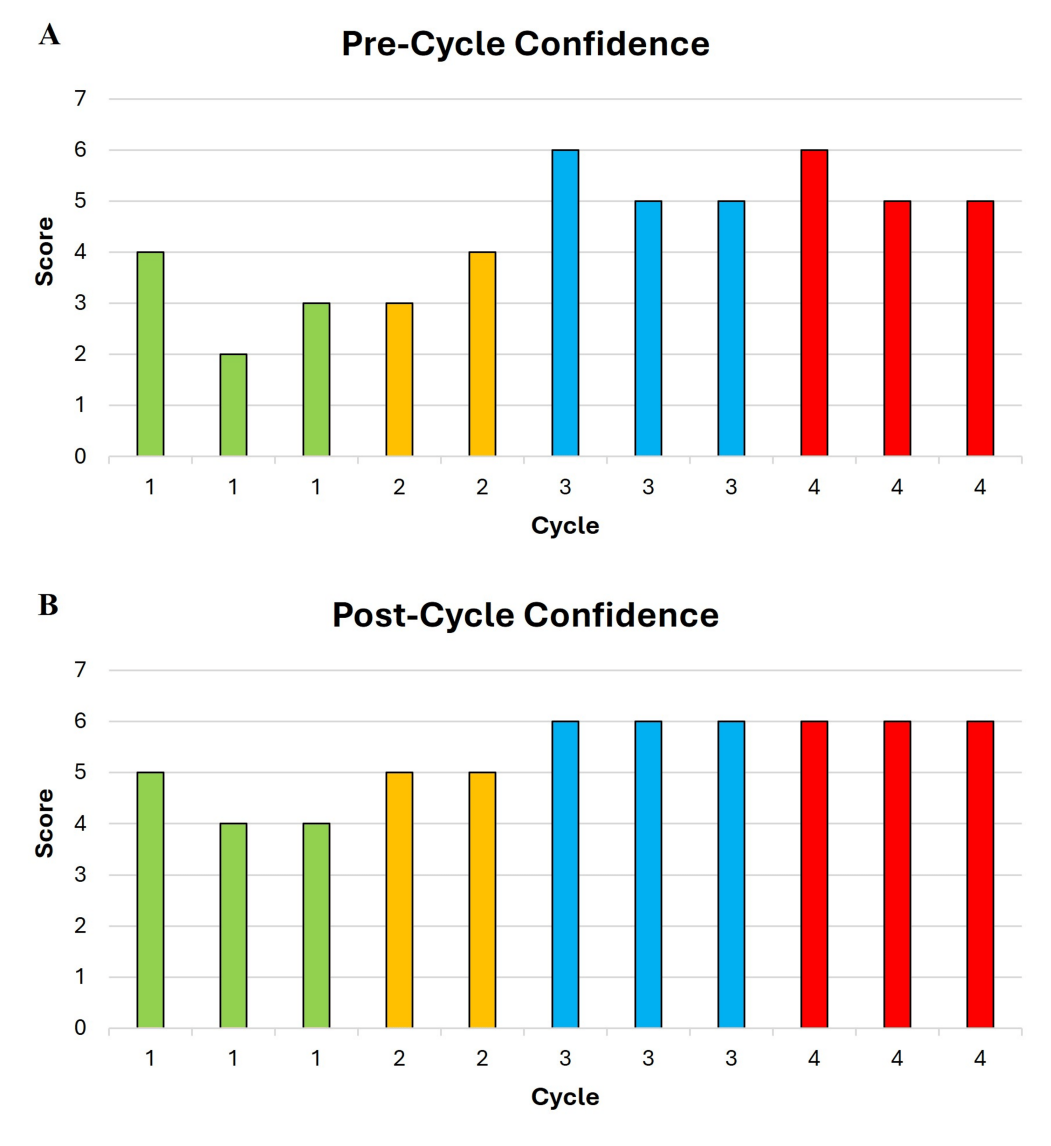
Resident confidence in chart review by cycle. Residents rated their confidence in their chart-review abilities (from 1 to 7) before (A) and after (B) each chart-review cycle. Each column represents one resident response and is arranged from lowest to highest score.

**Figure 6:**
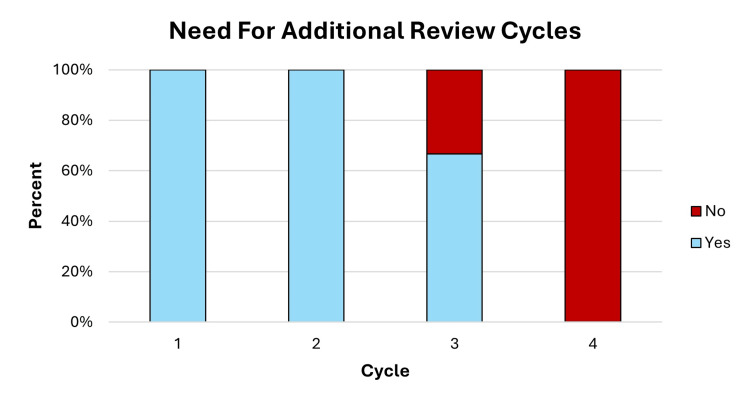
Resident need for additional cycles of chart review.

Resident confidence in chart review was queried with each cycle (Figures 5A, 5B). Pre-cycle confidence appeared to increase from cycles 1 to 3: mean scores 3 (2-4), 3.5 (3-4), 5.3 (5-6), 5.3 (5-6), in cycles 1 to 4, respectively (Figure 5A). Similarly, post-cycle confidence in chart review appeared to increase from cycles 1 to 3: mean score 4.3 (4-5), 5 (5), 6 (6), 6 (6), in cycles 1 to 4, respectively (Figure 5B). For each cycle, the increase in mean pre- and post-cycle confidence scores was 1.3, 1.5, 0.7, and 0.7, respectively. With each cycle, residents were also asked whether they felt additional cycles of chart review were needed. The perceived need for additional chart review cycles decreased over time - 100% of residents noted the need for additional cycles of peer review after cycles 1 and 2, compared with 66% after cycle 3, and 0% after cycle 4 (Figure 6).

**Figure 7:**
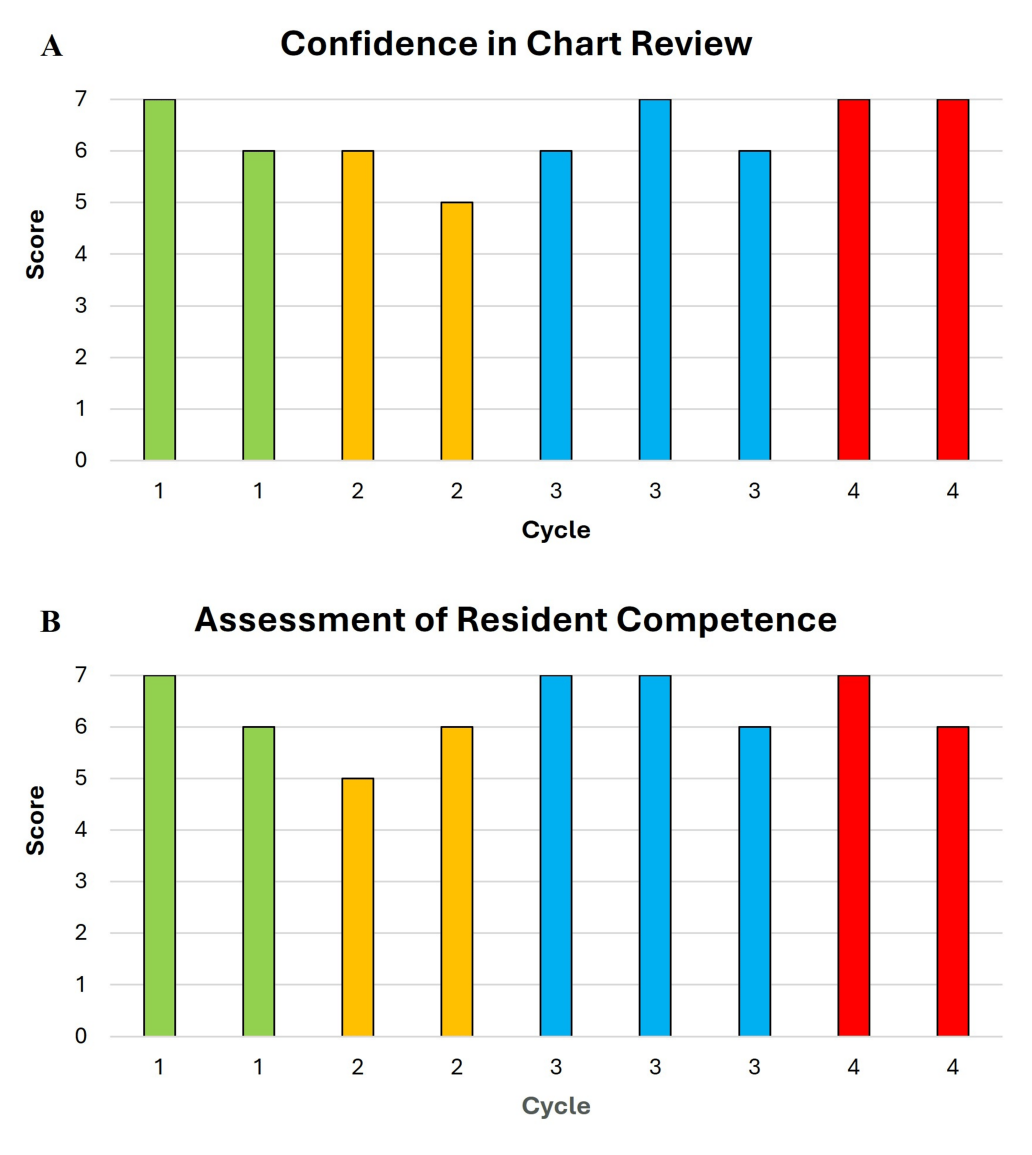
Attending perceptions on chart review. Attendings rated their confidence (from 1 to 7) in their chart-review abilities (A) and the competence of the resident they were paired with for each cycle (B). Each column represents one attending response and is arranged from lowest to highest score.

Attending perceptions were also assessed at the end of each chart review cycle (Figures 7A, 7B). Mean scores for attendings’ confidence in chart-review abilities were 6.5 (6-7), 5.5 (5-6), 6.3 (6-7), and 7 (7) across cycles 1-4, respectively (Figure 7A). Attending mentors graded resident competence in chart review at the end of each cycle from 1 (not at all ready) to 7 (ready) for independent chart review (Figure 7B). Mean competence scores were 6.5 (6-7), 5.5 (5-7), 6.7 (5-7), and 6.5 (6-7) for cycles 1-4, respectively. All seven scores were for chart reviews on either genitourinary or gastrointestinal malignancies.

Resident confidence in case review pre- and post- pilot PARRP

**Figure 8:**
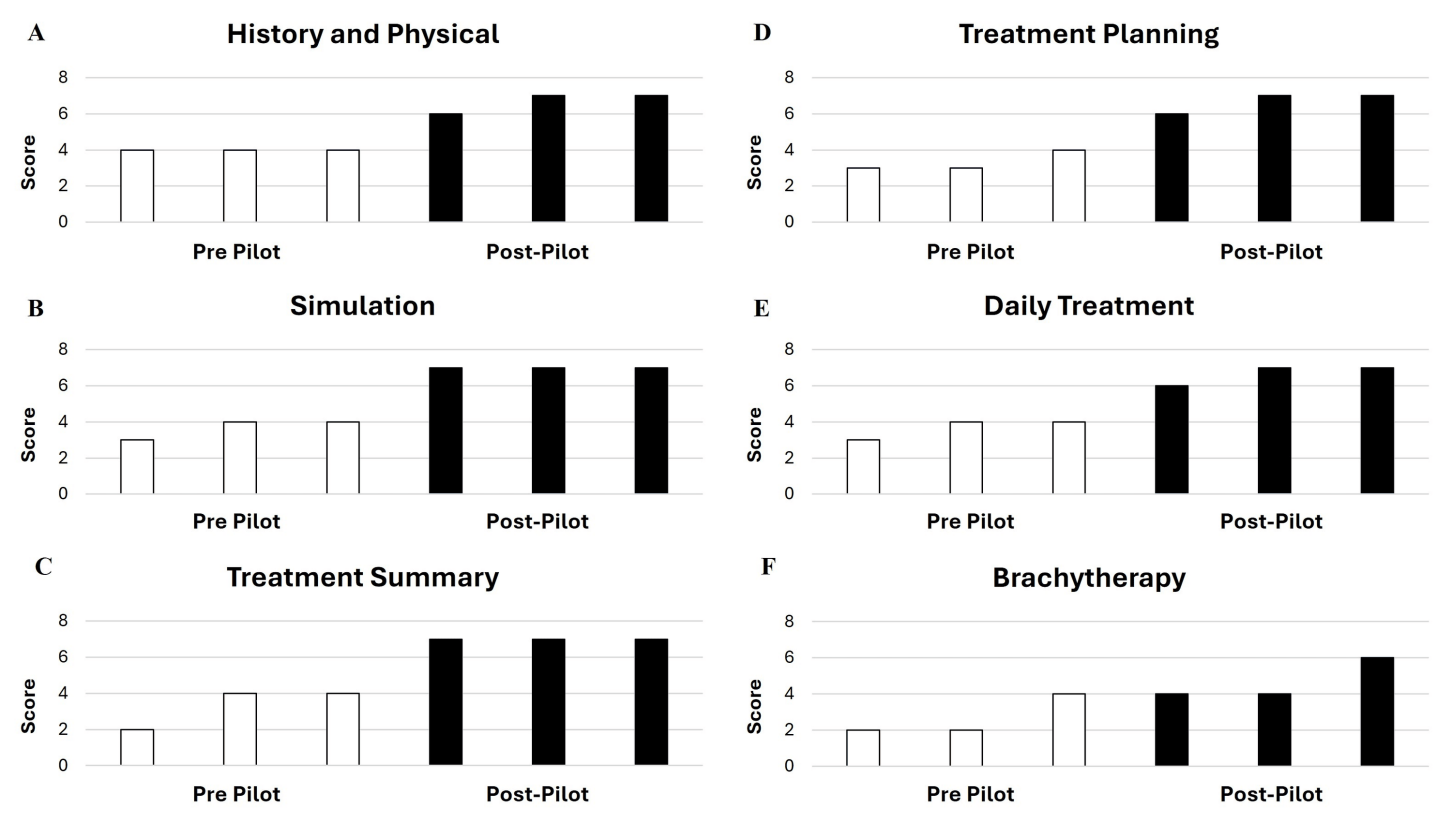
Resident confidence in evaluating components of chart review: pre- and post-PARRP pilot. Residents rated their confidence (from 1 to 7) in evaluating documentation of history and physical (A), simulation (B), treatment summary (C), treatment planning (D), daily treatment (E), and brachytherapy (F) before and after the PARRP pilot. Each column represents one resident response and is arranged from lowest to highest score. PARRP: Practice Accreditation Resident Reviewer Program

**Figure 9:**
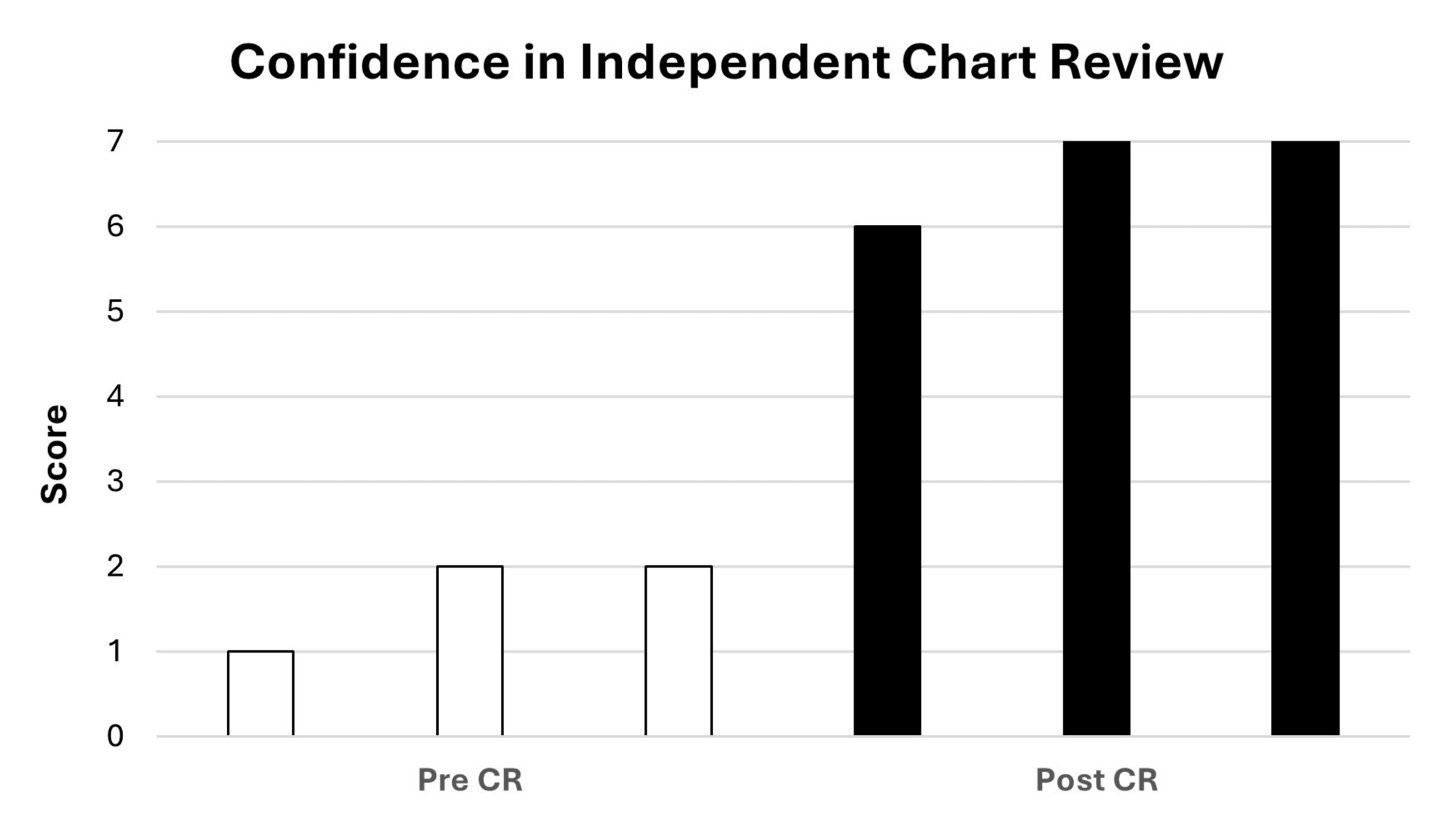
Resident confidence in independent chart review: pre- and post-PARRP pilot. Residents rated their overall confidence (from 1 to 7) in independently reviewing cases for practice accreditation before and after the PARRP pilot. Each column represents one resident response and is arranged from lowest to highest score. PARRP: Practice Accreditation Resident Reviewer Program

Figures 8A-8F summarize residents’ confidence in evaluating different components of the accreditation chart review. Across all domains, resident confidence in chart review improved after the pilot experience was completed. Mean pre- and post- pilot PARRP confidence scores were 4 (4) versus 6.7 (6-7) for history and physical documentation (Figure 8A); 3.7 (3-4) versus 7 (7) for simulation documentation (Figure 8B); 3.3 (2-4) versus 7 (7) for treatment summary documentation (Figure 8C); 3.3 (3-4) versus 6.7 (6-7) for treatment planning documentation (Figure 8D); 3.7 (3-4) versus 6.7 (6-7) for daily treatment documentation (Figure 8E); and 2.7 (2-4) versus 4.7 (4-6) for brachytherapy documentation (Figure 8F). Residents’ overall confidence in independent chart review improved from 1.7 (1-2) to 6.7 (6-7) before and after the pilot experience, respectively (Figure 9).

## Discussion

The PARRP was designed as a structured mentorship program to provide RO residents with additional experience in PSQI. This appears to be the first program to integrate RO residents as junior reviewers within an RO practice accreditation program. From this pilot program, we found that completion of PARRP led to an increase in confidence in multiple aspects of chart documentation review, including the history and physical, simulation, and treatment summary. Throughout the program, most feedback meetings lasted more than 30 minutes, and residents’ satisfaction with the meetings remained high with each chart review cycle.

The feedback meetings within PARRP provided an opportunity for structured mentorship. Mentorship programs within RO often utilize longitudinal dyads as they are commonly focused on career development [8]. PARRP, which focused on developing a specific skill set, utilized a multiple-dyad approach. This allowed residents to learn from different mentors with each chart review cycle. This approach is not unlike that used to train residents and fellows in peer review for academic publications. Both the American Society of Clinical Oncology and the American Society of Radiation Oncology have mentorship programs within their academic journals to teach peer review [9-13]. For example, in the Journal of Practical Radiation Oncology’s Review Apprentice Program, resident reviewers are paired with editors based on the disease site of interest [12]. After successful completion of five satisfactory reviews, residents can join the journal as reviewers [14].

The PARRP also provides an opportunity to enhance graduate medical education within RO. PSQI training is an integral part of graduate medical education in RO. ACGME guidelines stipulate that RO residency programs must provide training in PSQI [1]. Throughout residency, the resident's knowledge on this topic is rated and reported to the ACGME: competence in PSQI ranges from level 1 (novice) to level 5 (expert), with the goal of reaching level 4 by the time of residency graduation [2]. While residents at level 1 are expected to demonstrate knowledge of QI methodologies and metrics, those at level 5 are expected to create and implement QI initiatives at the institutional or community level.

How a resident achieves competence in PSQI is left at the discretion of the residency program. From a 2018 survey, RO and medical physics program directors reported that their programs used a variety of methods to teach PSQI, including morbidity and mortality conferences and safety/QI projects and activities [4]. While the majority of medical program directors believed residents were adequately prepared to meet PSQI expectations of clinical practice, RO residents report a different perspective [4]. For example, Spraker et al. found that only 40% of residents felt their PSQI training was adequate [3], while Fogh and Pawlicki reported low resident satisfaction with PSQI education [15].

Using a mentorship program to teach PSQI has several potential advantages. In this pilot program, feedback meetings were structured, and satisfaction with the meetings remained high with each subsequent review cycle. This is consistent with a prior review of RO mentorship programs that found that structured mentorship programs, compared to informal programs, were associated with higher satisfaction and increased confidence [8]. Additionally, the number of review cycles needed to improve confidence in independent chart review was small. By the end of cycle 4, none of the residents felt additional rounds of chart review were necessary. This is similar to a prior study of resident reviewer development in the Journal of Otolaryngology-Head and Neck Surgery [16]. Here, three mentored reviews were identified as the ideal minimum requirement for education in manuscript peer review.

The pilot PARRP experience had limitations. Primarily, the overall number of participants was small - three residents and four attending ROs. As a result, formal tests of statistical significance were not utilized, and results are meant to be hypothesis-driven. Additionally, the surveys focused on satisfaction and confidence with the program and did not formally assess resident knowledge of practice accreditation. While these data do not provide definitive conclusions regarding the use of structured mentorship for teaching PSQI in RO, they will inform the design of the next iteration of the PARRP. We plan to open the program nationally to include more residents and attending participants.

## Conclusions

The pilot PARRP demonstrated that a structured mentorship program in chart review can improve resident understanding of multiple aspects of practice accreditation. Satisfaction with feedback meetings remained high throughout the pilot program. A total of four cycles of review appears to be sufficient to achieve confidence in documentation review. Expanding the PARRP nationally may provide RO residents with an additional means to gain exposure to PSQI standards.

## Appendices

All study surveys

**Table 3:** Resident pre-study survey. This table was created by the authors of this study.

Item in the questionnaire	Scale
1. What year of residency training are you?	PGY-4, PGY-5, other (please describe)
2. What gender do you identify as?	Male, female, non-binary, prefer not to say, other (please describe)
3. Have you received formal training in patient safety and quality improvement during your radiation oncology residency?	No, yes
4. If yes, please describe type of training received.	Answer (write in response)
5. Were you familiar with practice accreditation prior to this program?	No, yes
6. Have you performed chart review for practice accreditation previously?	
7. How confident do you feel in your ability to review history & physical documentation?	1-7
8. How confident do you feel in your ability to review simulation documentation?	
9. How confident do you feel in your ability to review treatment planning documentation?	
10. How confident do you feel in your ability to review daily treatment documentation?	
11. How confident do you feel in your ability to review treatment summary documentation?	
12. How confident do you feel in your ability to review brachytherapy documentation?	
13. How prepared are you to review charts independently for a practice accreditation program?	
14. Do you feel you would benefit from mentoring in chart review for practice accreditation?	

**Table 4:** Resident assessment form. This table was created by the authors of this study.

Item in the questionnaire	Scale
1. Prior to this review cycle, how confident did you feel in your chart-review abilities?	1-7
2. Excluding the current cycle, how many cycles of chart review have you performed?	0, 1, 2, 3, 4, 5, 6 or more
3. How many charts did you review for this review cycle?	0, 1, 2, 3, 4, 5 or more
4. How long (in minutes) did you spend to review individual charts?	5, 10, 15, 20, 30, 40, 50, or more
5. How long (in minutes) did it take you to review all charts for this review cycle?	30 or less, 31-60, 61-120, 180 or more
6. How many days elapsed between receiving charts for review and submitting scores to your mentor for evaluation?	1, 2, 3, 4, 5, 6, 7
7. How many days elapsed between submitting scores to your mentor and the feedback meeting?	3 or less, 4-7, 8-10, 11-14, more than 14 days
8. How long (in minutes) did the feedback meeting with your mentor last?	<10, 10-30, 31-60, 60, or more
9. Did you find utility in the feedback meeting?	1-7
10. What is something(s) you learned from the feedback meeting?	Answer (write in response)
11. What is something you would change about the feedback meeting?	
12. How satisfied are you with the interaction with your mentor during this cycle?	1-7
13. After the feedback meeting, how confident do you feel in your chart-review abilities?	
14. After the feedback meeting, do you feel that additional cycles will improve your confidence in chart review?	No, yes

**Table 5:** Faculty assessment form. This table was created by the authors of this study.

Item in the questionnaire	Scale
1. Excluding your time as a resident, how many years have you been in practice as a radiation oncologist?	3-5, 6-10, 11-20, 20, or more
2. How many years have you been a reviewer for the ACRO accreditation program?	1 year or less, 2-5. 6-10, 11, or more years
3. How confident do you feel in your chart-review abilities?	1-7
4. In your day-to-day practice, how often do you interact with radiation oncology residents?	Never, rarely, occasionally, frequently
5. Prior to this review cycle, had you mentored residents as part of the Resident Chart Review Program?	No, yes
6. How many residents have you mentored as part of the Resident Chart Review Program?	0, 1, 2, 3, 4, 5, or more
7. How many charts did you review for this review cycle?	
8. How long (in minutes) did you spend to review each individual chart?	5, 10, 15, 20, 30, 40, 50, or more
9. How long (in minutes) did it take you to review all charts for this review cycle?	30 or less, 31-60, 61-120, 180, or more
10. Do you think the feedback meeting was useful for the resident?	1-7
11. What is something you would change about the feedback meeting?	Answer (write in response)
12. Following the feedback meeting, how would you categorize the resident’s competency in chart review for accreditation purposes?	1-7

**Table 6:** Resident post-study survey. This table was created by the authors of this study.

Item in the questionnaire	Scale
1. What year of residency training are you?	PGY-4, PGY-5, other (please describe)
2. What gender do you identify as?	Male, female, non-binary, prefer not to say, other (please describe)
3. How many cycles of chart review did you perform?	0, 1, 2, 3, 4, 5, 6, or more
4. How many charts (total) did you review for as part of this program?	Answer (write in response)
5. At the end of the Resident Chart Review Program, how confident do you feel in your ability to review history & physical documentation?	1-7
6. At the end of the Resident Chart Review Program, how confident do you feel in your ability to review simulation documentation?	
7. At the end of the Resident Chart Review Program, how confident do you feel in your ability to review treatment planning documentation?	
8. At the end of the Resident Chart Review Program, how confident do you feel in your ability to review daily treatment documentation?	
9. At the end of the Resident Chart Review Program, how confident do you feel in your ability to review treatment summary documentation?	
10. At the end of the Resident Chart Review Program, how confident do you feel in your ability to review brachytherapy documentation?	
11. At the end of the Resident Chart Review Program, how prepared are you to review charts independently for a practice accreditation program?	
12. How useful were the feedback meetings with your mentor?	
13. The feedback meetings improved my chart-review abilities.	
14. Did the Resident Chart Review Program improve your understanding of the practice accreditation process?	
15. Did the Resident Chart Review Program improve your understanding of quality care standards in radiation oncology?	
16. Would you be willing to serve as a reviewer for a practice accreditation program in the future?	No, yes
17. Would you recommend the ACRO Resident Chart Review Program to other radiation oncology residents?	Not at all, possibly, yes-definitely
18. What is something the Resident Chart Review Program did well?	Answer (write in response)
19. What is something the Resident Chart Review Program could improve?	
